# Identification of epidemiological risk factors for spotty liver disease in cage-free layer flocks in houses with fully slatted flooring in Australia

**DOI:** 10.1016/j.psj.2023.103139

**Published:** 2023-09-21

**Authors:** Yuanshuo K. Gao, Mini Singh, Wendy I. Muir, Michael Kotiw, Peter J. Groves

**Affiliations:** ⁎ Sydney School of Veterinary Science, Faculty of Science, The University of Sydney, Sydney, Australia; † School of Life and Environmental Sciences, Faculty of Science, The University of Sydney, Sydney, Australia; ‡ School of Health and Wellbeing, University of Southern Queensland, Toowoomba, Australia

**Keywords:** spotty liver disease, cage-free production, epidemiology, risk factors

## Abstract

Spotty liver disease (**SLD**) is recognized to be caused by infection with *Campylobacter hepaticus* in adult layer hens farmed in cage-free environments. SLD is an emerging disease as cage-free egg production increases in popularity in response to desires for improved welfare of poultry. Outbreaks of SLD are frequently experienced around peak egg production in flocks, commonly between 25 and 40 wk of age. The disease becomes manifest with increased exposure and access of the birds to the feces of the flock. This study follows from a previous epidemiological survey of free-range and barn flocks in Australia which identified the presence of a scratch area within the laying house as a major risk factor for the occurrence of SLD. However, that survey also observed SLD occurrence in 45% of houses with a fully slatted floor (no scratch area). The present study describes a further analytical survey aimed at identification of risk factors for SLD in houses with fully slatted flooring. A comprehensive questionnaire was completed for 49 cage-free flocks from point of lay until 40 wk of age across Australia, retrieving information on house design, bird breed, flock size, stocking densities, bird growth, and performance and the occurrence of SLD.

Multiple logistic regression model building was used to separate factors and identify important management factors that may be amenable to modify the occurrence of SLD in egg layers.

Key determinants of SLD identified from the analyses were that houses with mechanical ventilation (such as tunnel ventilation) have some protection from SLD and an increase of an extra 1 bird/m^2^ of nest space increased odds of occurrence of SLD by 1.172 times. A recommendation to not exceed 112 brown egg layer hens/m^2^ of nest space in naturally ventilated houses with a full slat floor was suggested. A delay in birds reaching 60% hen day production (**HD**) by 1 wk is suggested as a possible predictor for a subsequent outbreak of SLD.

## INTRODUCTION

Spotty liver disease (**SLD**) is a serious disease affecting adult egg-laying hens in commercial cage-free situations (free-range and barn lay systems). The condition is considered to be identical with an historic disease known as Vibrionic Hepatitis (Moore, 1958; Peckham, 1958) which disappeared with the introduction of cage layer systems. SLD has re-emerged with the increase in cage-free egg production in Australia. The systems of keeping laying hens by developed countries have been changing over the last 20 yr. Guyonnet (2022) reported that following the banning of conventional cages in Europe in 2012, housing systems for layers have changed variably, with some European states, such as France and Spain, adopting furnished cages as their predominant method of egg production. Others, such as Germany, chose to move to barn systems (indoor cage-free housing) while the United Kingdom has prompted more for free-range systems. Guyonnet (2022) also notes that in North America, colder conditions in Canada encouraged the adoption of furnished cages while the United States has moved toward cage-free aviary systems with the birds remaining inside the house. Aviary systems allow birds to have free access to a solid floor and although these do have slatted areas, they all essentially have a scratch area. In Asia and Latin America however, conventional cages still predominate (Guyonnet, 2022). Hence the Australian move toward predominantly free-range production is somewhat unique, mainly due to the more amenable year-round climate. Hence the relevance of the present project would be mostly applicable to free-range production in Australia and the United Kingdom. A meta-analysis by Weeks et al. (2016) described much higher mortality in free-range flocks in the United Kingdom compared to conventional cage systems although the reasons for this were not detailed, some of this could have been due to SLD. More recently Schuck-Paim et al. (2021) found that mortality rates in barn style cage-free production in Europe has reduced to levels similar to caged flocks, but this dealt only with raw mortality not cause-specific conditions and the differences between Australian and European operations and climate are substantial. A review by Bonnefous et al. (2022) noted that free-range management increases the risk of some bacterial, viral and parasitic diseases, and notes that *C. hepaticus* infections are increasing in Europe and hence the present study will be of relevance in regions where SLD is emerging.

The Australian commercial egg industry uses brown egg layers (Rhode Island breeds) almost exclusively. SLD is now known to be caused by an infective process with the recently identified and characterized bacteria, *Campylobacter hepaticus* (Crawshaw et al., 2015; Van et al., 2016; Phung et al., 2020) and the more recently identified *Campylobacter bilis* (Phung et al., 2022). Mortality rates during untreated SLD outbreaks can reach 10 to 15% and egg production may drop as much as 35% (Grimes and Reece, 2011; Courtice et al., 2018). The epidemiology of SLD is not well studied, with only some descriptive findings reported (Courtice et al., 2018). No analytical epidemiological studies on SLD have been published prior to Gao et al. (2023). Analytical studies aim to identify risk factors associated with the occurrence of a disease in a population of animals (Martin et al., 1987). This study follows on from information presented in a precursor study (Gao et al., 2023). The initial epidemiological survey identified that cage-free houses that included a scratch area were at much higher risk of developing SLD than those with the floor area fully covered by slats. However, almost half of the flocks with fully slatted flooring still developed clinical SLD. Hence a second survey examining flocks in lay in cage-free houses with fully slatted floors was undertaken to identify other factors which might modify the expression of SLD in this type of housing. The aim of the present study was to identify further factors that could be subject to intervention and thus allow a change in the risk of SLD. Factors that have a significant effect on a disease and that are under the control of farm management are termed “key determinants” (Martin et al., 1987).

## MATERIALS AND METHODS

The survey was conducted under the supervision of the Animal Ethics Committee of the University of Sydney (protocol number 2021/1898).

### Survey Design

Farms were sought to participate from those who participated in the original SLD epidemiological survey (Gao et al., 2023), from egg producers that attended seminars on SLD conducted by 2 of the authors which were organized by Australian Eggs Ltd. and from farms serviced by interested poultry veterinarians. All producers approached cooperated willingly as SLD is of high concern in the Australian cage-free egg industry.

A questionnaire was designed based on the preliminary findings of the first cross-sectional survey (Gao et al., 2023), with a heavy focus on shed infrastructure, resource availability, and bird production data. A copy of the survey questionnaire is included as supplementary information. The survey was carried out retrospectively during 2020 to 2021. The introduction of travel restrictions in Australia due to the COVID-19 pandemic disrupted the visitation plans for the survey. Interviews for flocks in New South Wales (**NSW**) were able to be carried out in person, whereas information for all interstate flocks was obtained by interview by phone or by online discussion. Data were collected retrospectively. An example of the questionnaire is included supplementary material.

All farms involved used birds reared on separate farms. All birds had beak treatment (infrared) administered by the hatchery (there are only 2 hatcheries producing commercial egg layers in Australia). Birds were all vaccinated against Marek's Disease, Newcastle Disease, Infectious Bronchitis, Fowl Pox, Infectious Laryngotracheitis, Avian Encephalomyelitis, Egg Drop Syndrome ’76, Fowl Cholera, and *Salmonella* Typhimurium at the hatchery and at the rearing farms under standard industry practices using Australian registered vaccines.

Participating flocks were categorized as “Cases” or “Controls.” The case definition used was that the flock experienced a rise in mortality and a decline in egg production associated with the occurrence of typical gross pathology of SLD: that is, multiple focal necrotic lesions (spots) in the liver, and a fibrinous perihepatitis possibly with icterus. SLD in all “Case” flocks was diagnosed by the veterinarian consulting to the particular farm. Collection of cloacal swabs, feces, or dust was only achieved on only 13 houses; 8 of which were classified as Controls and 5 as Cases. Of the 8 Control flocks, 7 returned dust samples which were positive for the presence of *C. hepaticus* and all 5 of the cases also had positive cloacal swab or dust samples by PCR (as developed by Van et al., 2017). As the survey was conducted retrospectively and most farm visits were made impossible due to strict government travel restrictions during the COVID-19 pandemic.

### Statistical Analyses

Data were transferred to MS-Excel and uploaded into JMP v16,1,0 (SAS Institute Inc, 2022, www.jmp.com) for analysis.

All survey variables were assessed in a univariate analysis using a contingency table analysis for categorical variables or Student *t* test for continuous variables with the Case or Control definition as the dependent variable. Any variable displaying a probability of an association being due to chance value of <0.20 (as suggested by Hosmer et al., 2013) was selected for further inclusion in any multivariate model building approach. This high probability value is used as a screening level for selection of possibly important factors the significance of which may be hidden within the complexities of the problem. Pearson χ^2^ or Fisher's exact 2-tailed tests (the latter when an expected value was <5) were used to assess probability of associations being due to chance. Continuous variables expressing a probability of being due to chance of <0.20 were selected for further analysis.

Multiple logistic regression is an iterative statistical test that allows assessment of the effect of factors after controlling for the presence of other factors, thus assisting in eliminating extraneous effects. It also allows assessment of confounding and interaction between factors (Dohoo et al., 2003; Hosmer et al., 2013). Confounding exists when “the results of 2 or more factors cannot be separated” from each other (Blood and Studdert, 1999). All selected variables were considered in a univariate logistic regression which is able to consider several levels of a variable (e.g., slat brands). Variables showing a statistical association with occurrence of clinical SLD were then added to a multivariate logistic regression analysis along lines suggested by Dohoo et al. (2003). Variables that showed a probability due to chance of <0.05 were then examined for confounding and then subjected to a forward stepwise analysis including interaction terms to identify the key factors. A final multivariate analysis was then performed to define the major factors involved in risk of SLD in fully slatted houses. Odds ratios (**Ω**) for significant variables were calculated from the regression coefficients (**β**) such that Ω = e^β^.

## RESULTS

A total of 49 flocks contributed to the survey. Of these, 20 flocks (41%) were categorized as “Cases” and 29 (59%) were regarded as “Controls.” Table 1 shows means, median, and range of values for a number of descriptive statistics. Table 1 also summarizes the extent of the effects of SLD in some of the farms which experienced the disease. Mean age of an SLD outbreak was 31 wk with a range of 22 to 47 wk of age. All flocks experiencing SLD were treated promptly by veterinarians so the full effects would have been modified. The duration of the disease was up to 7 wk and mortality on affected farms due to SLD ranged between 0 and 9% and depression in egg production varied between 0 and 14% over the outbreak periods.

**Table 1:** Descriptive statistics for houses included in the survey (*N* = 49).

Variable	Mean	Minimum	Median	Maximum
Date flock transferred to laying house	7 Jan 2021	9 Oct 2019	19 Feb 2021	28 Jan 2022
Age transferred to laying house (wk)	16.28	14	16.14	20.14
Stocking density (birds/m^2^)	10.14	7.44	10.50	12.11
Nest space (birds/m^2^)	116.99	58.30	118.77	195.36
Age first access to nest boxes (wk)	16.73	15.43	16.64	20.00
Range stocking density (birds/ha)	3497.4	1153.9	2222.2	9662.6
For SLD Case flocks (*N* = 20)^1^				
Age at SLD outbreak (wk)	30.97	22.29	31.21	47.00
Total percentage of flock lost due to SLD	4%	0%	4%	9%
Duration of mortality due to SLD (wk)	3.71	0	3.00	7.00
Percentage drop in hen day egg production during SLD	8%	0%	8%	14%

1 Medication was administered to all SLD-affected flocks, affecting mortality and egg production outcomes variably.

House ventilation system was flagged as of interest from the small sample number of fully slatted sheds examined in the previous survey (Gao et al., 2023). Out of 49 sheds enrolled in the present survey, 8 used mechanical ventilation control (tunnel ventilated) and the remaining 41 were of conventional, naturally ventilated design. This study's observations identified no Cases occurring in tunnel ventilated houses. Hence a tunnel ventilated fully slatted house was identified as a putative protective factor against SLD, although a strong conclusion here was limited by the low sample number. As was found in the previous survey (Gao et al., 2023), the zero cell value for Cases confounds all other factors within tunnel ventilated facilities. Hence only naturally ventilated houses could be considered further for the analysis. This reduced the sample size to 41. Layer breeds were not evenly distributed across the house ventilation types and only 1 layer breed was present in 39 of the naturally ventilated flocks. This distribution negates any breed comparison in this study as representation of other breeds was negligible.

Table 2 presents the remaining categorical variables in contingency tables identifying the number of naturally ventilated houses experiencing SLD (“Case”) or not (“Control”). Hosmer et al. (2013) recommend an initial selection of variables for further evaluation as those that the univariate analyses produce a probability of being due to chance as less than 0.25 or 0.20. The use of a *P* value of 0.05 at this early stage of the analytical process may not detect important variables which may be confounded or involved in interactions with other variables. Hence, based on *P* < 0.20 as a screening level, categorical variables were selected for further assessment, which included nest/slat brand, closure of nests at night, and nutritionist used. Only 4 flocks did not close their nests at night, making this variable difficult to interpret and this variable was not considered further.

**Table 2:** Categorical variables distributed across Case or Control sheds in naturally ventilated houses.

Variable	Level	No. Case houses	No. Control houses	χ^2^ or Fisher's exact 2-tailed test^1^*P*=
Season chicks hatched	Autumn	6	10	0.25
	Winter	6	3	
	Spring	5	5	
	Summer	1	5	
Age of house	Converted from older	7	6	0.50
	Purpose built	11	17	
Perches in rearing shed	Yes	7	9	0.80
	No	11	12	
Nest and slat brand^2^	A	7	13	**0.04**
	B	4	5	
	C	6	1	
	D	0	4	
	E	1	0	
Slat shape	Rectangular	5	3	0.34
	Square	6	5	
	Oval	7	13	
Lights type	Cool white	10	8	0.61
	Warm white	7	8	
Nests closed at night	Yes	16	17	**0.12**^1^
	No	0	4	
Nutritionist	A	8	8	**0.02**
	B	1	5	
	C	3	8	
	D	5	0	
Feed mill	W	8	8	0.37
	X	6	5	
	Y	3	8	
	Z	1	0	
Water additives used till 40 wk	Yes	4	5	0.91
	No	14	16	
Number of rations used in lay	1	12	13	0.76
	>1	6	8	
Perches in laying shed	Yes	14	14	0.48^1^
	No	4	7	

1 Pearson χ^2^ test but if an expected value was <5, Fisher's exact 2-tailed test was used. Bold *P* values indicate factors considered worthy of further statistical evaluation (*P* < 0.20).

2 A, Vencomatic; B, Roxell; C, Big Dutchman; D, Facco; E, Salmet.

Table 3 shows outcomes of Student *t* tests conducted using Case or Control as the dependent variable and all continuous variables measured in the study in naturally ventilated sheds. Tables 2 and 3 were used to select factors for further analysis if the probability of the association of being a Case was <0.20. There were some missing data points in many variables due to differences in the information that farmers generally record and the information that could be provided. Obtaining full records was hampered by travel restrictions due to COVID-19 during 2021, especially with regard to flocks outside of NSW. Continuous variables considered further included stocking density (birds/m^2^ useable floor space in shed), perch space in lay, nest space (birds/m^2^ of nest space), the age at which birds were first allowed to range (weeks), range density (birds /ha), age (weeks) at which the flock reached 5% hen day production (**HD**), age they reached 60% HD, the body weight of the birds at 60% HD, age at peak lay, and the number of weeks between transfer to the laying shed and the flock reaching peak HD production.

**Table 3:** Continuous variables distributed across Case or Control sheds in naturally ventilated houses.

Natural ventilation ONLY *N* = 41	Control	Case				Control	Case	
Student *t* tests, independent variables⁎	Mean	Mean	*t* value	df	*P*=	Valid *N*	Valid *N*	Total *N*
Age transferred (wk)	16.09	16.10	−0.03	39	0.974	23	18	41
Stocking density (birds/sq. m)	10.17	9.61	1.44	39	**0.158**	23	18	41
Perch space in lay (cm/bird)	45.63	86.51	−1.77	37	**0.084**	21	18	39
Total nest space on record (sq. m)	153.74	144.93	0.70	36	0.490	21	17	38
Nest space on record (birds/sq. m)	101.41	115.86	−2.44	36	**0.020**	21	17	38
Age of first access to nest boxes (wk)	16.63	16.57	0.21	35	0.836	21	16	37
First let out age (wk)	22.12	22.71	−0.69	32	0.497	17	17	34
Range size (ha)	7.06	5.65	1.07	36	0.291	21	17	38
Range density (birds/ha)	3232	4666	−1.59	36	**0.122**	21	17	38
Number of rations from arrival to 40 wk of age	1.87	1.61	0.82	39	0.416	23	18	41
Body weight at arrival (kg)	1.38	1.39	−0.34	38	0.735	23	17	40
Age at first egg (wk)	19.02	18.84	0.49	32	0.629	18	16	34
Age at 5–10% HD^1^ (wk)	19.65	20.16	−1.34	35	**0.190**	21	16	37
Age at 60% HD^1^	21.71	22.38	−1.59	34	**0.122**	20	16	36
Body weight at 60%HD^1^ (kg)	1.75	1.80	−2.06	28	**0.049**	17	13	30
Age at peak lay (wk)	28.86	27.55	0.86	34	0.394	19	17	36
Body weight at peak lay (kg)	1.85	1.90	−1.30	20	0.207	11	11	22
Time since transfer to 60% HD^1^ (wk)	5.40	6.16	−1.71	34	**0.097**	20	16	36
Body weight gain from transfer to 5–10% HD (g)	251	275	−0.72	29	0.477	18	13	31
Body weight gain from transfer to 60%HD (g)	341	418	−106.6	28	**0.042**	17	13	30
Body weight gain from transfer to Peak lay (g)	487	494	−0.19	20	0.854	11	11	22

Bold factors deemed worthy of further statistical evaluation (*P* < 0.20).

⁎ Continuous value variables.

1 HD, hen day production.

Several of these variables are autocorrelated (i.e., occurrence of 1 variable was strongly associated with the occurrence of another). Notably, factors related to bird age were correlated with body weights at that age, as birds continue to gain weight throughout lay: older birds are heavier. Five of the factors selected for further study fall under this association and data for all of them was incomplete as hen body weights were not always recorded every week. Dohoo et al. (2003) suggested a valid technique to deal with this type of autocorrelation would be to intuitively select 1 factor as representative of all of the related factors, based on the investigator's knowledge of the husbandry system and the relative *P* values of each variable with the dependent variable. In this case, the time since transfer until the flock reached 60% HD production was selected as the best representative, as this factor had the highest complete data set and the lowest *P* value of being due to chance in its association with the flock being a “Case.” Thus, this variable provides a representation of age at transfer, age at initiation of egg laying, and rapidity of the rise in egg production. This gave 8 remaining variables to further assess within naturally ventilated sheds.

Each identified variable was then entered into a univariate logistic regression model. For continuous variables, logistic regression evaluates the effect of a change of 1 unit on the likelihood of an effect on the dependent variable. The outcomes for the univariate logistical regression of each of the variables selected for further analysis are presented in Table 4. This analysis showed that nest/slat brand and nutritionist produced large standard errors giving unstable estimates of regression coefficient (**β**) and extreme odds ratios (**Ω**) between types, indicating that including them would make the model overfitted (Hosmer et al., 2013).

**Table 4:** Univariate logistic regression for selected factors for risk of SLD.

Variable	Level	β^1^	Standard error of β	Ω^2^	Wald's test	*P*=
Nest/slat brand^3^	C	1.60	737.7	11.14	4.19	0.38
	D	−16.39	1475.5	1.7 × 10^−7^		
	B	−0.41	737.7	1.49		
	E	16.01	2660.0	2.02 × 10^7^		
	A	Reference	Unstable			
Nutritionist	A	−5.26	608.2	5.00	2.53	0.47
	B	−3.65	608.2	1.47 × 10^8^		
	C	Reference	Unstable			
	D	13.55	1824	1.875		
Perch space in lay	cm/bird	0.008	0.0046	1.01	2.94	0.087
Range density	1000 birds/ha	0.0002	0.0001	1.00	2.37	0.12
Stocking density in shed	Birds/m^2^	−0.381	0.270	0.68	2.00	0.157
Nest space	Birds/m^2^	0.086	0.057	1.09	2.297	0.130
Time from transfer to 60% HD	Weeks	0.476	0.305	1.61	2.437	0.119

1 Logistic regression coefficient.

2 Odds ratio for risk of SLD.

3 A, Vencomatic; B, Roxell; C, Big Dutchman; D, Facco; E, Salmet.

The associations of nest and slat brand and nutritionist delivered unstable coefficient estimates with extreme standard error values (Table 4) and were removed from further consideration. Perch space in lay, stocking density in the house, range density, nest space density, and time from transfer to 60% HD produced statistically valid regression coefficients and odds ratios of the association between the variables and the SLD outcome. These remaining terms were entered into a multiple logistic regression model (Table 5). The multiple logistic regression model assessed each variable, controlling for the presence of each other variable in the model. Using this approach, perch space in lay and range density exhibited nonsignificant associations and were removed from further consideration in the model. The remaining 3 variables were examined for confounding by sequentially entering each variable into a multiple logistic regression model and evaluating major changes made to the estimate of the regression coefficient for each. Variables were considered likely to be confounded if their regression coefficient changed by more than 30% by the addition of another variable to the model (Dohoo et al., 2003).

**Table 5:** Multivariate logistic regression for valid factors for risk of SLD.

Variable	Level	β^1^	Standard error of β	Ω^2^	Wald's test	*P*=
Intercept		−28.499	15.74			
Perch space in lay	cm/bird	0.007	0.013	1.01	0.30	0.581
Range density	1000 birds/m^2^	0.340	0.331	1.40	1.06	0.304
Stocking density in shed	Birds/m^2^	−1.532	0.608	0.216	6.34	**0.012**
Nest space density	Birds/m^2^	6.628	3.026	756.3	4.80	**0.029**
Time from transfer to 60% HD	Weeks	0.664	0.428	1.94	2.40	**0.121**

1 Logistic regression coefficient.

2 Odds ratio for risk of SLD.

**Figure 1:**
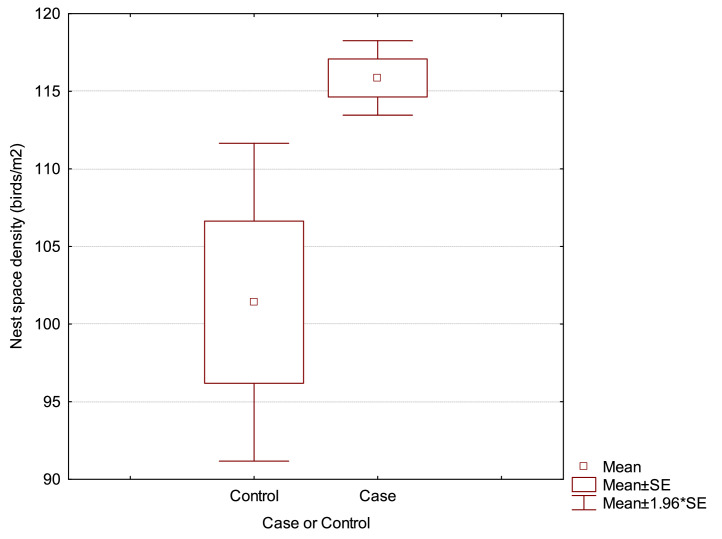
Box and whisker plot of distribution of birds/m^2^ of nest space for Control and Case flocks for SLD occurrence. Bars denote ± 95% confidence intervals.

Stocking density was found to be strongly confounded by both the addition of nest space (41% change in coefficient estimate) and also by time from transfer to 60% HD (66% change) to the model. Nest space and transfer time to 60% HD were not confounded with each other. Nest space and transfer time to 60% HD showed acceptable fit of the models. Hence the final model for assessment of risk factors for SLD in naturally ventilated fully slatted-floor sheds contained only nest space (birds/m^2^) and time (weeks) from transfer to the laying quarters until the flock reached 60% HD. A term for the interaction between these remaining 2 variables was added to the model. The final model is shown in Table 6. The means and 95% confidence limits for nest space allowance for Control and Case flocks is shown in Figure 1. The main findings resulting from these analyses were that a delay in birds reaching 60% HD by 1 wk increased the odds of occurrence of SLD by 5.736 times. These analyses indicated that an increase of an extra 1 bird/m^2^ of nest space increased odds of occurrence of SLD by 1.172 times). Hence, using the means, the increase in the risk of SLD occurring when the number of birds per nest m^2^ is increased from 101 to 115 is (1.172)^14^ = 9.23 (i.e., a 923% increase in risk) (Dohoo et al., 2003). The sample means here are an estimate of the actual population mean and the 95% confidence limits for the Control mean were between 90.5 and 112 birds/m^2^ of nest space, while the Case 95% confidence limits were from 113.3 to 118.5 birds/m^2^.

**Table 6:** Final multivariate logistic regression analysis of identified variables associated with SLD.

			Likelihood ratio tests				Ω confidence limits	
Term	β^1^	SE	χ^2^	*P*=	LogWorth^2^	Ω^3^	Lower 95%	Upper 95%
Intercept	−28.396	15.97						
Time from transfer to 60% HD^4^ (wk)	1.747	1.042	3.841	0.050	1.301	5.736	1.000	63.645
Nest space (birds/m^2^)	0.158	0.100	5.910	0.015	1.822	1.172	1.017	1.471
Nest space (birds/m^2^) × Transfer to 60% HD (wk)	−0.136	0.102	2.079	0.149	0.826	0.873	0.694	1.043

Whole model test								
Model	−LogLikelihood	DF	χ^2^	*P*=		*R*^2^	**0.2401**	
Difference	5.937	3	11.873	0.008				
Full	18.793					BIC^5^	51.922	
Reduced	24.731					Observations	36	

1 Logistic regression coefficient.

2 A statistic based on χ^2^ to evaluate a contribution due to chance in the full model (=(−log_10_(*P* value)). Higher values signify more contribution to the model.

3 Odds ratio for risk of SLD.

4 HD, hen day % production.

5 Bayesian information criteria—minimum BIC used for selection of best model fit.

## DISCUSSION

An earlier cross-sectional survey of SLD in cage-free egg layer flocks in Australia (Gao et al., 2023) identified the presence of fully slatted flooring in the house as a partially protective factor against the disease, compared to houses which gave access to the solid floor area (scratch area) within the house. The present study was undertaken to examine other factors that may be involved in determining the likelihood of an outbreak of SLD in flocks which had the floor completely covered by slats. A broad questionnaire was administered across 49 cage-free (free range or barn style) flocks across Australia and the results examined through a multiple logistic regression analysis technique.

Hosmer et al. (2013) suggest a cutoff of probability due to chance association between putative factors and the outcome variable well above the usual value of <0.05, as variation, confounding and interaction in field data can often obscure associations which may actually be significant risk factors. These relational problems often produce aberrant analyses. These interrelationships needed to be assessed to determine the most meaningful associations between factor presence and the disease outcome. Entering each variable into a logistic regression model can reveal erroneous issues where extreme values of standard errors lead to biased or unstable associations as observed in this study between stocking density with regard to floor space in the house and nest space availability. Some variables had minimal valid numbers for either Case or Control (e.g., nest closure at night) and could not be validly analyzed.

The data indicated that shed with tunnel ventilation have some protection against the occurrence of SLD. Courtice (2022) reported that the severity of SLD can be much reduced if the shed temperature can be reduced by 8°C at the start of an outbreak. This intervention will obviously be more possible in mechanically ventilated sheds than in those relying on open sided ventilation (termed “natural ventilation” in the present report). The finding in the present study would suggest that protection against an outbreak of SLD may be possible if temperature control is optimal before an outbreak develops. Other possible factors could also contribute to the ameliorating value of a tunnel ventilation system. Tunnel ventilation systems provide forced air movement through the length of the house, achieving cooling by the wind chill factor of air speed, but this also introduces more fresh air, increases air flow and helps remove noxious gasses such as ammonia and carbon dioxide. The more effective fresh air movement may also possibly reduce SLD transmission and promote general health.

Further, this observation of the protective role of tunnel ventilation for SLD was consistent with putative findings from the previous study (Gao et al., 2023). The absence of clinical SLD in the present study in tunnel ventilated sheds meant that further analyses could only be considered across naturally ventilated sheds within the database.

In continuing the analysis for naturally ventilated houses only, identified putative factors were then subject to multiple logistic regression techniques to draw out the most likely contributors and develop a parsimonious model for risk of SLD. Once the confounders were removed, acceptable odds ratios were developed for the remaining important variables.

Nest space (number of birds/m^2^ of nest) emerged as the most important remaining factor (*P* = 0.015) in the model for naturally ventilated sheds within this survey. An odds ratio of 1.172 for higher number of birds/m^2^ of nest space was estimated. That is, for every increase of 1 bird/m^2^ of nest space, the risk of SLD rises by 1.17 times. This was consistent with the preliminary findings from the previous study (Gao et al., 2023). The mean nest space for Control flocks was 101.4 birds/m^2^ of nest space compared with 115.9 for flocks which experienced SLD (Cases). The suggested maximum nest space allocation suggested by a free-range brown egg layer breed management manual (HLB, 2021) is 120 birds/m^2^ in colony nests. Bessei et al. (2013) noted that competition for nest space, especially early in lay, may be a considerable stressor for commercial hens and that brown egg layers have a higher requirement for nest space than do white egg layer breeds as brown egg breeds spend more time in the nests. If hens compete more for nest space, this could be a trigger for the expression of SLD in flocks. The current study would lead to a recommendation that it would be advisable to not exceed 112 birds/m^2^ of nest space (the upper 95% confidence limit for unaffected flocks) in naturally ventilated houses with fully slatted flooring to avoid a higher risk of SLD occurring.

There were also associations of several variables involving the age of the birds to begin and continue into egg production and an association with the occurrence of SLD in the flock. These included bird age at 5% HD (“point of lay”), age at 60% HD, body weight at 60% HD, time (weeks) from the birds’ arrival in the laying shed (“transfer”) until the flock reached 60% HD, body weight at 60% HD and weight gain from transfer to 60% HD. There was missing data for some of these factors as farmers do not consistently weigh birds at each week. These factors however are linked, as weight increases with age. In all the factors considered in this group, the mean age for Control flocks was about 0.5 to 0.6 wk younger at point of lay and at 60% HD and in terms of the time from bird transfer to the layer quarters until they reached 60% HD, than for Case flocks. In this situation, 1 variable was chosen to represent the correlated variables (Dohoo et al., 2003). The time from transfer to 60% HD was selected in this instance as that variable contained the least number of missing values. The association of this factor, the time between transfer to layer quarters and when the flock reached 60% HD production, approached statistical significance (*P* = 0.050) in its association with the occurrence of SLD in naturally ventilated flocks with fully slatted flooring. It is not clear whether the observed significant association of birds reaching 60% HD production later (Controls at 21.71 wk compared with Cases at 22.38 wk: a difference of 0.67 wk or 4.7 d) with a higher risk of occurrence of SLD represents a cause or an effect. Arguably flocks coming into lay slightly later may be more likely to experience SLD resulting from physiological effects on their development and maturity, or, conversely, flocks being affected by early subclinical infection with SLD may have their onset of lay delayed. It is not possible to determine which of these is a critical component for SLD occurrence from the data available in this study. What can be understood, however, is that flocks that are later and slower to come into lay (say point of lay at 20 wk rather than 19 wk of age), may be more at risk of a subsequent SLD outbreak. In this sense, the observed delay in onset and continuance of lay may be a predictor for SLD.

The confounding between stocking density and nest space allowance was interesting. While higher numbers of birds per nest space area was a risk factor for SLD, higher numbers of birds/m^2^ of available floor space was protective (odds ratio <1.0). The Hyline Brown breed management manual recommends 7 to 9 birds/m^2^ of useable space in free-range houses (HLB, 2021). The stocking density observed in this study had a mean of 9.92 birds/m^2^ with a range of 7.44 to 11.92 birds/m^2^ of useable floor space. There was no correlation between nest space and shed stocking density (correlation coefficient, *r* = −0.05, *P* = 0.766). However, nest space (birds/m^2^) was strongly negatively correlated with the ratio of total nest space (m^2^) in the shed to the total available floor space (m^2^) (*r* = −0.92, *P* < 0.0001). From this it is obvious that where nest space makes up a greater proportion of the shed area, there will be a lower number of birds/m^2^ of nest space but a higher number of birds/m^2^ of available floor space. This would explain the confounding between the 2 measures of nest space and floor space. Statistically, the measure of fit (Deviance) of the model of stocking density as a risk factor for SLD showed that the stocking density parameter did not fit the data well and thus nest space was more significant and is the risk factor identified here for SLD.

## CONCLUSIONS

In layer sheds that have a full slat coverage of the floor area, a further key determinant of the risk for SLD occurrence in the flock is having a natural ventilation system (i.e., an open sided house with curtains or shutters and only air circulation fans inside the house). Tunnel ventilation systems may provide some protective effect against SLD.

In houses with fully slatted floors and a natural ventilation system, increasing the number of birds/m^2^ of nest space results in an increased risk of SLD occurrence. Based on the available data for this survey, for every increase of 1 extra bird/m^2^ nest space increases the risk of SLD by 1.172 times (i.e., by 17.2%). Highest recommended nest space allocation developed from this survey data would be 112 birds/m^2^ of nest space for colony nest systems.

A delay in the onset of egg production and a later achievement of 60% HD production by about 5 d may be predictive of a later SLD occurrence in the flock.

As ventilation system and number of birds placed in the house/m^2^ of nest space are amenable to modification by management, these factors can be declared as key determinants (Martin et al., 1987) for SLD in fully slatted houses.

## Appendix Supplementary materials

Image, application 1
