# Supplementary material for: Identification of epidemiological risk factors for spotty liver disease in cage-free layer flocks in houses with fully slatted flooring in Australia
Source: Poult Sci. 2023 Sep 21;102(12):103139. doi: 10.1016/j.psj.2023.103139 (PMC10685297; doi:10.1016/j.psj.2023.103139)
Supplement: Supplementary file 1 [file mmc1.pdf]

**Date:**	
**Company Name:**	

	**Company name**				
	**Layer farm name**				
	**Shed number**				
	**SLD occurance (Y/N)**				
REARING FARM					
	Date hatched				
	Breed				
	Hatchery				
	Perches in rearing? (Y/N)				
	Ventilation in rearing shed: tunnel or natural				
LAYING FARM					
	Location of laying farm (Suburb)				
	Date transferred				
	Age transferred (wk)				
	Number of birds transferred				
**Shed set up**	Shed Type (conventional vs aviary free-range)				
	Total usable space (m2)				
	Stocking density (birds/m2)				
**Slat**	Slat brand				
	Slat material (e.g. plastic)				
	Is the slat cleaned during the batch? (Y/N)				
	If yes, how?				
	Is the slat cleaned between batches? (Y/N)				
	If yes, how?				
**Perch**	Perches in lay (Y/N)				

	Total perch length available (m)				
	Perch space in lay (cm/bird)				
**Platform**	Any platform(s) in shed? (Y/N)				
	Total platform area available (m2)				
	Platform space (birds/m2)				
	Is the platform cleaned during batch (Y/N)?				
	If yes, how?				
	Is the platform cleaned between batches (Y/N)?				
	If yes, how?				
**Feeding System**	Feeder type (chain or pan)				
	Total feed chain length (m)				
	Feed space - Chain (birds per m)				
	Total number of pans (if applicable)				
	Pan brand				
	Feed space - Pan (birds per pan)				
**Drinking system**	Drinker type				
	Total nipples in shed				
	Drinker space - nipple (birds per nipple)				
	Drinker space - bell (birds per bell)				
**Ventilation**	Ventilation in laying farm: tunnel or natural				
**Lighting**	Light colour (warm or cool white)				
**Nesting**	Nest box brand				
	Total nest space avaialble (m2)				
	Nest space (birds/m2)				
	Age of first access to nest boxes (wk)				
	Night closure (to prevent bird access) (Y/N)				

	Nest box cleaning during batch (Y/N)				
	If yes, how?				
	Nest box cleaning between batches (Y/N)				
	If yes, how?				
**Range**	First let out age (wk)				
	Range size (ha)				
	Range density (birds/ha)				
	Soil type				
**Feed and Water**	Nutritionist				
	Feedmill				
	Number of rations from arrival to 40 weeks of age				
	Ration 1 start age (wk)				
	Ration 2 start age (wk)				
	Ration 3 start age (wk)				
	Ration 4 start age (wk)				
	Ration 5 start age (wk)				
	Any in feed additives in lay till 40 weeks of age (Y/N)				
	Feed Additive 1 (if applicable)				
	Feed Additive 2 (if applicable)				
	Feed Additive 3 (if applicable)				
	Feed Additive 4 (if applicable)				
	Any water additives in lay till 40 weeks of age (Y/N)				
	Water Additive 1 (if applicable)				
	Water Additive 2 (if applicable)				
	Water Additive 3 (if applicable)				
	Water Additive 4 (if applicable)				
**Arrival**	Body weight at arrival (kg)				
**First Egg**	Age at first egg (wk)				

**5% HD**	Age at 5% HD (wk)				
	Feed intake at 5%HD (g/bird/day)				
	Body weight at 5%HD				
**60%HD**	Age at 60% HD				
	Feed intake at 60%HD (g/bird/day)				
	Body weight at 60%HD				
**Peak lay**	Age at peak lay (wk)				
	Peak lay %HD				
	Feed intake at peak lay (g/bird/day)				
	Body weight at peak lay (kg)				
**SLD outbreak (if applicable)**	Age at SLD outbreak (wk)				
	Feed intake at SLD (g/bird/day)				
	Body weight at SLD (kg)				
	Lowest %HD during SLD				
	How long did				
	Highest daily mort% during SLD				
**40 weeks**	%HD at 40 weeks				
	Feed intake at 40wks (g/bird/day)				
	Body weight at 40wks (kg)				
	Total mortality at 40wks				
**Egg production**	Any problem with floor eggs? (Y/N)				
	Floor egg% at peak lay				
	Age occurred				
	Duration of floor egg issue (>2%) (days)				
**Disease**	Other disease other than SLD (Y/N)				
	Age of disease occurance (wk)				
	Any antibiotic treatment not for SLD?				
**SLD SPECIFIC**	Did SLD occur this batch (Y/N)				
	Has SLD reoccured so far?				
	Age of outbreak 1 (wk)				

	How long did the first outbreak last?				
	Age of outbreak 2 (wk, if applicable)				
	How long did the second outbreak last?				
	SLD diagnosis based on (select all that apply):				
	Clinical signs (depression)				
	Gross pathology (white spots)				
	Histopathology				
	PCR				
	Culture				
	By treatment				
	Not sure				
	Was this flock treated with antibiotics for SLD (Y/N)				
	What antibiotic was used?				
	Other antibiotic used, please specify:				
	Range access prior to SLD outbreak?				
	Any comments on SLD outbreak of this flock not covered above?				
